# An IoT-Aware Solution to Support Governments in Air Pollution Monitoring Based on the Combination of Real-Time Data and Citizen Feedback

**DOI:** 10.3390/s22031000

**Published:** 2022-01-27

**Authors:** Teodoro Montanaro, Ilaria Sergi, Matteo Basile, Luca Mainetti, Luigi Patrono

**Affiliations:** Department of Engineering for Innovation, University of Salento, Via Monteroni snc, 73100 Lecce, Italy; teodoro.montanaro@unisalento.it (T.M.); ilaria.sergi@unisalento.it (I.S.); matteo.basile@studenti.unisalento.it (M.B.); luca.mainetti@unisalento.it (L.M.)

**Keywords:** Internet of Things, machine learning, smart cities, government facilities, air pollution, traffic monitoring

## Abstract

One of the main concerns of the last century is regarding the air pollution and its effects caused on human health. Its impact is particularly evident in cities and urban areas where governments are trying to mitigate its effects. Although different solutions have been already proposed, citizens continue to report bad conditions in the areas in which they live. This paper proposes a solution to support governments in monitoring the city pollution through the combination of user feedbacks/reports and real-time data acquired through dedicated mobile IoT sensors dynamically re-located by government officials to verify the reported conditions of specific areas. The mobile devices leverage on dedicated sensors to monitor the air quality and capture main roads traffic conditions through machine learning techniques. The system exposes a mobile application and a website to support the collection of citizens’ reports and show gathered data to both institutions and end-users. A proof-of-concept of the proposed solution has been prototyped in a medium-sized university campus. Both the performance and functional validation have demonstrated the feasibility and the effectiveness of the system and allowed the definition of some lessons learned, as well as future works.

## 1. Introduction

Air pollution is one of the most worrying issues of the last decades due to the negative effects that it could cause to the body. In fact, as declared by Gupta et al. [1], it can cause different health problems for humans like stroke, heart diseases, lung cancer, and respiratory diseases. The problem acquires more importance also due to the declarations of the World Health Organization (WHO), which claimed that more than 80% of people live in urban areas in which the air quality level frequently exceeds the WHO limits, as also reported by Becnel et al. [2].

Consequently, the interest of the research community in monitoring environmental parameters in modern cities has rapidly grown, as also confirmed by Spandana et al. [3], and new solutions have been proposed to mitigate or solve the caused issues.

One of most important innovative paradigms that is strongly motivating and supporting such an effort in monitoring and mitigating the effects of pollution in cities is the Internet of Things (IoT) concept [4]. Thanks to its broad network of sensors (such as connected thermometers, weather stations, or surveillance cameras) it is revolutionizing almost every aspect of our daily lives.

The adoption of Information and Communication Technologies (ICT) and, specifically, IoT solutions in the city context has been referred as “Smart City” environment [5]. This concept has emerged to “exploit the ICT in making better use of the public resources, increase the quality of services offered to the citizens and, in turn, the quality of life in urban areas, while reducing the operational costs of the public administrations” [6].

Its benefits have been demonstrated by several research articles (e.g., the literature reviews published by Mohsin et al. [7] and Arroub et al. [8]) that have exploited IoT to enhance various situations and, obviously, it was also applied in monitoring city air pollution, as demonstrated by different works and literature reviews [1,3,8,9,10].

Nonetheless, the level of pollution in a city or in a specific zone of a city can rapidly vary during a certain period due to different factors and events. For instance, Boso et al. [11] detected the pollution increasing in a particular period in the city of Temuco, Chile. As revealed, in fact, due to the restrictions imposed by the COVID-19 pandemic, citizens that were forced to remain at home increased their usage of wood-burning stoves to warm their house and, consequently, generated more pollution.

For this reason, it is important for government entities to leverage on real time systems and platforms to allow rapid and prompt interventions in case of dangerous situations but also accurate evaluations of monitored data to schedule long-term operations for improving the quality of citizens lives. To this aim, as supported by Doran et al. [12], communities could benefit from both automatic detection of environment parameters data, but also from crowdsensing systems able to “involve people in the planning process” [13] by collecting their feedback to then notify strange or dangerous situations to city officials that could, finally, intervene to monitor or to mitigate problems in the short, medium, and long period.

For this reason, based on the analysis of the state of the art that demonstrates the lack of a solution to support local governments in monitoring city conditions through the combination of user feedback and real-time data acquired through dedicated mobile IoT sensors, the present paper proposes a solution for city officials and citizens to support (a) the collection of users’ reports about perceived increasing pollution, (b) the rapid and dynamic re-location of mobile sensors to effectively reveal the updated situation of a reported area, (c) the decision-making process for new interventions in that area, and (d) the final discussion on possible new public green areas. Thanks to the developed system, city officials can exploit a centralized interface on which all the data and feedback are collected and, by its consultation they can promptly and easily have an overview of the current situation of the city and correlate, for instance, the information related to the traffic with the one related to pollution. In addition, the possibility of receiving notifications and dynamically re-locate mobile sensors to investigate reported anomaly situations allow them to intervene without the common delay.

The remainder of the paper is organized as follows. Section 2 analyzes related works, while Section 3 reports two scenarios used to identify requirements and needs. Section 4 and Section 5 describe, respectively, the proposed architecture and the implementation details that could guide future or similar works. Section 6 reports a first functional validation of the proposed system. In Section 7 a first performance analysis of some system parameters is summarized. Section 8 presents a discussion in which our solution is compared with exposed related works. Finally, Section 9 concludes the paper and sketches future works.

## 2. Related Works

Different works can be found in the literature, addressing the needs of monitoring environmental parameters in modern cities through IoT technologies as also demonstrated by different literature reviews [8,9,10]. However, they are mainly concentrated on the benefits of the technologies themselves and only a few of them combine distributed mobile monitoring sensors and systems with citizens opinions/feedbacks in the detection and effects’ mitigation processes.

A first interesting contribution that exploits distributed mobile sensors but without user feedbacks is the one proposed by Spandana et Shanmughasundram [3]. The authors use an inexpensive and effective system to monitor the air pollution level in particular areas, focusing on the selection of low-power sensors and mobile systems moved in specific locations to monitor the nearby area. Preliminary results demonstrated the feasibility and the efficacy of the system in monitoring the environment through low-cost devices.

In addition, Becnel et al. [2], present a system that exploits a group of low-cost pollution monitoring stations operating as single nodes that periodically collect airborne pollution data. In this case, the approach is a bit different from the one used in our work. In fact, the authors preferred to distribute various sensors throughout the city, instead of dynamically re-locate them in interesting zones and/or exploit end-users’ feedback in the selection of the areas to be monitored, as we propose in this article.

A similar work by Patil et al. [14] presents an environmental monitoring system based on Wireless Sensor Networks (WSNs) that use low-cost sensors to collect air pollutant information in different zones of a monitored city. Captured data is transferred to a cloud back-end where they are analyzed and exposed to the end user through an Android application that is able to report the pollution level in the various locations of city.

Garzon et al. [15], on the other hand, leverages on the assumption that it is possible to divide the city into different zones, each of which with a different level of atmospheric pollution. Therefore, the authors developed a system that sends a notification to a user when he/she is entering an area with a high pollution (i.e., with a dangerous pollution rate).

All the related works presented above are focused on the exploitation of distributed mobile sensors throughout different areas of the city for the monitoring of environmental parameters without any exploitation of user feedback; however, some other works adopt the opposite approach, concentrating the effort on the use of user “inputs” for monitoring the city without focusing the attention on the sensing IoT-aware technologies.

White et al. [16], for instance, propose a urban digital twin that allows users to report feedback on planned changes of the city. Their paper proposes a public digital twin of an area in Dublin city and illustrates how this model could be used for urban planning of skylines and green space.

R. Cecconi et al. [17], instead, propose a system to involve citizens in the urban degradation survey process and to support strategic decisions of the public administration, through a streamlined process. A web-based decision-making platform exploiting community participation was developed as a modular system involving both technical surveys on physical urban degradation and perceived urban degradation assessment provided by citizens. Outputs are thematic maps, used by the public administration to make strategic decisions on how to invest funds for the improvement of urban deficiencies.

Ertiö et al. [18] examine three cases of citizen-initiated online platforms aimed at investigating what happens when communities use technologies in specific geographic location contexts. The authors focus on understanding how citizens can actively participate in planning and designing the communities through the use of technologies. The research is based on secondary data collected from the online and social media sites of the three case studies.

Among the few works that combine distributed mobile monitoring sensors with user feedbacks, the one that is most similar to our approach is the one proposed by Rinaldi et al. [19]. The authors describe the early stage of implementation of a system that leverages on the dialogue between building and users. The developed Smart Campus Demonstrator is equipped with sensors to monitor and control comfort, indoor air quality and HVAC parameters. In addition, it includes the behavioral perspective linking the users’ mobile application to the framework and achieve a bidirectional interaction between the built environment and the social landscape. The feedback of the users was only related to the data collected through users smartphone’s sensors and was used to activate actuators to mitigate uncomfortable situations. On the other hand, in our work we consider real user feedback in terms of answers received in proposed surveys and we exploit it to suggest new interventions to city officials.

Tagliabue et al. [20] propose a framework to support the real-time evaluation and control of a wide range of sustainability criteria within a building. The paper adopts a user-centered point of view and exploits a combined approach based on Digital Twin (DT) and Internet of Things (IoT). The framework allows both the monitoring of buildings’ status through dedicated sensors and the evaluation of user feedback to determine the right strategies to optimize the trade-off with renewable energy production. A pilot building in the University of Brescia (Italy) was used to test the framework with some sample applications. The building accommodated the daily activities of the students by interacting with the sensorized asset monitoring indoor comfort and air quality conditions as well as the energy behavior of the building. The approach described by Tagliabue et al. is similar to the one we present in this article, but it is focused on indoor monitoring of buildings and does not exploit mobile sensors to be moved in case of need by city officials.

### Monitoring Sensors

Within the present work different sensors have been used to monitor the air pollution and the traffic of specific area of the city. The sensors and the devices to be used were selected among the ones most frequently used in literature. For this reason, the present paragraph summarizes the inspiring works from which the choice was based.

Environmental pollution monitoring is often performed by sophisticated and well-known tools that are generally huge and expensive. For instance, the various electrochemical sensors used by Tan et al. [21] to measure different gases present in the air have a fairly high average cost (about USD 2000). Despite the very high guaranteed quality and reliability of such sensors, due to the continuous renovation of the treated topics and the consequent renovation of the needed material, researchers usually prefer to select cheaper sensors to be replicated or easily substituted in case of necessity. To this aim, Yi et al. [22] present in their work the technological low-cost and small sized alternatives for monitoring air pollution, with technological progress. As demonstrated, although the quality of the measures is not the same as the more expensive ones, their use allows to reduce costs and, as needed in the presented paper, expand the monitored area. This is the main reason why, in this work, we chose to use low-cost sensors to monitor the air quality. A similar choice was taken by Rani et al. [23] who chose the Hanwei MQ Sensor (a low-cost device: its cost was about USD 10) for the real-time monitoring of the air quality.

In the present work, with the need of choosing sensors to be spread around the city, and the additional requirement of providing as much Mobile Smart Monitoring Device as possible, we decided to use low-cost sensors despite their reliability. In particular, the sensors MQ135 [24,25], MQ7 [26,27], and DHT11 [28,29] were used to measure the temperature, humidity, and gas values (carbon monoxide, carbon dioxide, oxidized ammonia). Meanwhile, the YL-83 rain sensor [30,31] was adopted to detect rain. In addition, the Raspberry Pi Camera [32,33] was selected to acquire pictures of city streets and zones, and, consequently, in case of need, estimate the traffic conditions by contacting the Cloud Server.

Such sensors are usually connected to a controller that can obtain as input the measured data, elaborate it, and send it to a Server for further elaboration or storage. Most of the research projects related to environmental monitoring usually use the Raspberry PI [34] as the controller interface due to the simplicity of its programming and the low-cost characteristic. Therefore, as also done by Riddhika et al. [35] and Zhang et al. [36], in the present work the Raspberry Pi 4 Model B board was selected to implement such a controller.

## 3. Scenario

The design and the implementation of the mobile solution proposed in this paper were guided by the two scenarios presented in this section that describe two real situations in which the solution could be valuable for city officials and citizens. The scenarios were inspired by the state of the art analysis and were designed with the aim of having two use cases that cover the points of view of the two main stakeholders of the proposed solution. In the following subsections a summary of each scenario is proposed to guide the extraction of the functional requirements reported at the end of this section.

### 3.1. Scenario 1: Citizen Point of View

In the first scenario, the point of view of a citizen is presented. It foresees the common behavior of a woman living in a medium-sized city chosen for the excellent quality of the air and the really low level of pollution. We can assume that she likes to monitor ambient parameters such as the temperature, the humidity, and the CO${}_{2}$ level through dedicated sensors installed on the roof of her house and also through some weather stations installed by the city local government officials exposing real-time data on a dedicated website. With such systems, we can suppose that the user suddenly notices a strange gas emission (e.g., by observing a column of smoke emitted by the nearest shopping center) in the area in which she lives and, by consulting the services (the one provided by the local government and the personal one), she notices that there is a discrepancy in the data shown in the government website and the one acquired through her service. Therefore, she would like to report the problem to the city government officials, but the only way she has is to send an email that, probably, will remain unread for a long time, or to contact a local newspaper to report the situation as soon as possible (before the center hides it).

### 3.2. Scenario 2: City Officials Point of View

In the second scenario, the point of view of the local government of the city (e.g., the same city in which the user of the first scenario lives) is presented. We can suppose, in this case, that the officials installed new monitoring weather stations, one for each district, able to monitor both weather conditions and pollution levels. The officials daily access the system user interface to check if everything is going well and to identify areas in which it could be necessary to invest new funds to, for example, plant new trees. Within such a context, an official would be interested in becoming aware of the situation described in the previous scenario in which a shopping center emits strange gases but the only instrument that a citizen can exploit to report the problem is the email. In addition, the official does not have any tool or device to easily and rapidly install in the interested area to monitor the situation and investigate if it is a recurring problem or only the problem of one day. Furthermore, another situation that is commonly notified by citizens and that the city official cannot monitor is the one related to the correlation among the traffic and the pollution in a specific area. In fact, although the installed systems are spread all over the city, it is not possible to install them in every corner or street of the city and, therefore, if a user sends a notification about, for example, the growing traffic in an area caused by the opening of a new shop, the official can only contact a new company for installing a new system in the interested area.

### 3.3. Functional Requirements

The previous scenarios were designed as representatives of the realistic functionalities that should be offered by the proposed solution.

Starting from the motivation presented in the Introduction and the Related Works sections and inspired by the scenarios, Table 1 summarizes the main requirements that guided our work. They are mainly concentrated on two needs. First, as discussed by White et al. [16], R. Cecconi et al. [17], and Ertiö et al. [18], the citizen feedback on city conditions is greatly appreciated by both the local government officials that deal with their daily duties and the citizens that would like to express their opinion. Second, as demonstrated by Becnel et al. [2], Shanmughasundram [3], Patil et al. [14], and Garzon et al. [15], another important element highly appreciated by city officers is the possibility of using IoT sensors and devices for monitoring pollution and visualizing an overview of their city condition.

### 3.4. Non-Functional Requirements

In addition to the Functional Requirements, Table 2 presents the transversal requirements that the system should satisfy to be effective and useful.

## 4. System Architecture

**Figure 1:**
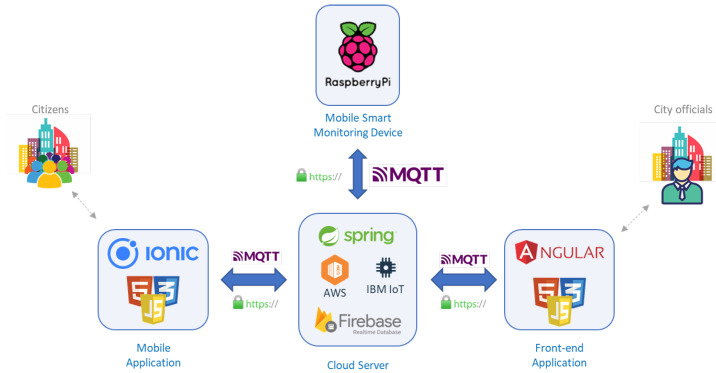
System architecture of the proposed solution.

Figure 1 shows the proposed system architecture, designed to satisfy all the reported requirements and serve the two main stakeholders: citizens and city officials. For space reasons, in the Figure some implementation details are anticipated although they will be discussed in the next sections.

The shown architecture is composed of four main parts: a Mobile Application (App), a Mobile Smart Monitoring Device, a Cloud Server, and a Front-end Application.

The Mobile App is the component that is completely dedicated to the citizens and allows citizens to send to the Cloud Server different types of reports and feedbacks about the city in which they live and its conditions. Thus, when a citizen would like to report something strange in the city or in the zone of the city in which she lives, she can fill a pre-defined survey for warning the city officials. Each feedback can be linked to some additional information such as a picture of the revealed problem, or the GPS position of the user or the coordinates of the area in which the problem is reported. As an additional feature, the app is also responsible for showing notifications to the citizen when a sent feedback/report is opened by a local government official. To guarantee privacy, two different types of report can be sent by the citizens: anonymous or protected. Anonymous reports can forward information that the citizen does not want to be associated to her, while protected reports are the ones sent by a user that wants to be recognized but, obviously, only by the city government officials (e.g., a report containing personal information, like the precise address) and can be sent only if the user was previously authenticated. To this aim, the Mobile App exploits an authentication process.

Another component of the architecture is the Cloud Server. It acts as a big gateway that puts each component in communication with each other. As a first example, in fact, all the reports sent by the Mobile Application are forwarded to the Cloud Server that is responsible for their storage and their sharing with other modules. Therefore, the Cloud Server is able to guarantee: (a) the infrastructures (i.e., some dedicated REST APIs and publish/subscribe solutions) to receive and share the data received by both the Mobile Smart Monitoring Devices and the Mobile Applications; (b) the ecosystem to receive and share the reports received by the Mobile Applications; (c) the infrastructure to receive and forward to all the interested components, the alerts generated by each component; (d) support for storing the data and reports in a database; (e) a specific Machine Learning Service to help the Mobile Smart Monitoring Device in the detection of the traffic and, in particular, in returning the number of people, truck, motorcycles and pedestrians detected in sent images; (f) the infrastructure to invoke commands from one component to the others (e.g., reset the alert number threshold in the Mobile Smart Monitoring Device); (g) the infrastructure for defining interventions to solve a reported or detected situation; and (h) the infrastructure to manage operators actions to solve defined interventions.

Another important component is the Front-end Application through which the data received from the Mobile Smart Monitoring Device and the reports received by the Mobile Application are exposed to city officials. In fact, the Front-end Application is the graphical component that provides the interface to let officials benefit from proposed services. It allows to: (a) receive notifications about reports sent by citizens; (b) access fine grained data about a city zone; (c) monitor other situations, like traffic, in a specific zone; (d) access historical data to, for instance, evaluate the possibility of planting new trees; (e) set and reset threshold for the generation of the alerts messages; and (f) manage interventions and guide operators during the duties associated to an intervention. Moreover, the Front-end Application is responsible for the visualization of the data acquired through the Mobile Smart Monitoring Devices distributed in strategic points of the city.

In fact, the final, but most important, component that mainly differentiates our work with respect to existing ones is the Mobile Smart Monitoring Device. By managing and analyzing the citizen feedback, the city officials can distribute various Mobile Smart Monitoring Devices in different strategic areas of the city to monitor the areas interested by the received report. Such devices are designed to collect environment related data such as temperature, gas values (e.g., CO, CO${}_{2}$, AMM), humidity, rain presence, and traffic conditions, and store them in a database. The smartness feature of the device allows a first interpretation of the data on the edge, and the generation of notifications in case of detected anomalies.

As will be more investigated in the following sections, the design of all these components addressed all the functional requirements described in the previous paragraphs. In addition, although most of the transversal Non-functional requirements are mostly dependent on the implementation of each component and the selected tools, programming languages, operating systems, and protocols used, the design phase took great care of them in order to design an effective and useful architecture. The Security requirement, for instance, has to be treated in the design phase as demonstrated by McManus [37]. In fact, the most used technique for respecting this requirement is the so called “Security by Design” approach that suggests to design software products and capabilities to be foundationally secure. These principles have been applied in the devise of the presented architecture that, thanks to its intrinsic modularity nature, allows the introduction and the continuous update of all the strategies needed to respect the security requirements. Furthermore, the Usability requirement is also connected to the modular intrinsic characteristic of the designed architecture. In fact, the Front-end was designed as an independent module that can be continuosly updated to satisfy changing user needs without affecting the other components.

Finally, the modularity also satisfies the Scalability requirement by allowing to independently duplicate each module in case of growing amount of work.

## 5. Implementation Details

With the aim of verifying the feasibility of the proposed architecture and the effectiveness of the proposed solution to help officials in dynamically monitoring specific areas of the city after receiving reports from citizen, a prototype of the proposed architecture has been developed. A summary of all the tools and technologies adopted for the implementation is reported in the already presented Figure 1.

**Figure 2:**
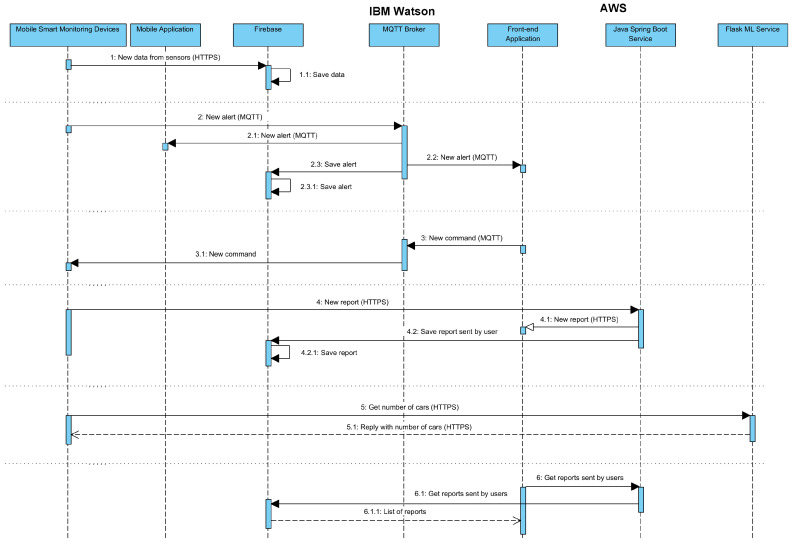
Sequence Diagram of all the interactions among different components.

In addition, Figure 2 reports the most important interactions among the components and will be used as a guide in the following paragraphs for explaining the message exchanges.

The main component of the architecture is the Cloud Server that, as already depicted in the previous section, is responsible for different services. In this first prototype, these services are distributed among different platforms. In the following subsections, we present, in turn, the technologies selected for the storage; the ones selected for the sharing of information, alerts, and commands; the ones selected for sending reports filled by the user; and the ones selected for the Machine Learning service for the traffic detection.

The storage of the information is entrusted to the Firebase platform [38] that exposes a service to receive data through dedicated APIs, stores such data in a dedicated internal database (Firestore Database) and, additionally, shares such a data with registered devices or applications. Therefore, as shown in Figure 2 by interactions n. 1 and n. 2, every time the Mobile Smart Monitoring Devices acquires a new data, it is directly sent to the Firebase component that saves such data into its database. In addition, as shown by interactions n. 3 and n. 7, the Firebase platform is also used to store the history of all the generated alerts and the estimation of the traffic performed through the Flask ML Service that will be presented in the next paragraphs. Nonetheless, due to the revealed latency of the Firebase service with respect to other well-known solutions, the MQTT protocol was selected as the secondary channel for sharing alerts. MQTT is a publish/subscribe-based messaging protocol especially suited for low-resource embedded systems and for applications in which the network bandwidth is limited [39]. Due to its characteristics, it was selected for our case, in which the alerts and commands should arrive in a short time in all circumstances, e.g., also when the Mobile Smart Monitoring Device is moved in a part of the city in which the connection is not stable. Within this prototype, the IBM Watson IoT Platform [40] was selected as the free service on which the MQTT Broker, the central most important element of an MQTT network, has been deployed. The format used to share the data on this Broker is the JavaScript Object Notation (JSON) format, one of the most used in these circumstances. Two main MQTT topics were, thus, designed for the communications: “commands” to change the behavior of any component (e.g., to set the alert threshold in the Mobile Smart Monitoring Device) and “alerts” for sending and receiving all the alerts (e.g., high temperature). Consequently, as shown in Figure 2 in interactions n. 3 and n. 4, the MQTT protocol is used to publish a new alert or a new command. Thus, when a new alert is published, the registered elements, i.e., the Mobile Application, the Front-end application, and the Firebase component receive the notification and perform consequent actions. Furthermore, similarly, when a new command arrives, a notification is also sent to the Mobile Smart Monitoring Devices.

Finally, the AWS platform was selected for the services responsible for: (a) receiving reports from citizens, managing operators, assigning reports to operators, and defining the surveys; and (b) detecting the traffic through the dedicated Machine Learning algorithm. To this aim, two dedicated applications were developed, the first being a Java Spring Boot [41] service that exposes the REST APIs, some of which are shown as an example in Table 3 (some minor resources are not reported for lack of space) to manage the reports and store them into the Firebase database. Consequently, some REST resources are dedicated to gather reports and manage boundary information.

Meanwhile, the second application is a Python Flask [42] service that exposes the REST APIs shown in Table 4 (also in this case the minor resources are not reported) to obtain an estimation of the number of people, trucks, motorcycles, and pedestrians revealed in a received image.

The application is based on the fourth version of the well-known “YOLO” Machine Learning algorithm [43] pre-trained through the public Coco Dataset [44] and implemented exploiting the Tensorflow2 library [45]. YOLO is a Machine Learning model based on Neural Network for the detection of real time objects. It is, in fact, defined as a general-purpose object detector that is increasingly used by various works due to its good accuracy and high speed. As a Machine Learning algorithm, for its usage it is usually needed to train the model through a dataset. The fourth version of such model (YOLOv4) allows, instead, to use a pre-trained model and, in our case, as already anticipated, the model pre-trained through the Coco Dataset was used within a Flask service. Specifically, this pre-trained model has been integrated into the flask code importing the yolov4v2.0.2 Python library and instantiating the YOLOv4 object. This object exposes various methods that allow to predict which are the objects present within an image received in input. The Coco Dataset is composed of 80 classes of objects, consequently, the pre-trained model is able to predict up to 80 kind of objects including the vehicles present in a road, as needed in our case. Therefore, for the purpose of this paper, the model was used to recognize vehicles and/or pedestrians in the stream of images received by the camera. As a summary, in fact, the traffic monitoring service starts the acquisition of the images from the camera, sends each image to the YOLO model, and receives a feedback on the presence of vehicles or pedestrians in the monitored road. The interactions with the ML component are represented by the connections n. 5 and 5.1 of the Figure 2. The JWT (Json Web Token) [46] mechanism is used to secure all the REST APIs and authenticate the requests. This characteristic guarantees high reliability and availability of the exposed services. In addition, the selection of well-known tools and technologies like the consolidated Cloud solution proposed by AWS, guarantees the satisfaction of all the transversal requirements discussed in the previous sections. In fact, AWS was born exactly to simplify the work of IT employees in respecting the discussed requirements. Therefore, the RN5 constraint related to the Scalability is guaranteed by the AWS provider that declares to provide vertical scalability of its machines.

**Figure 3:**
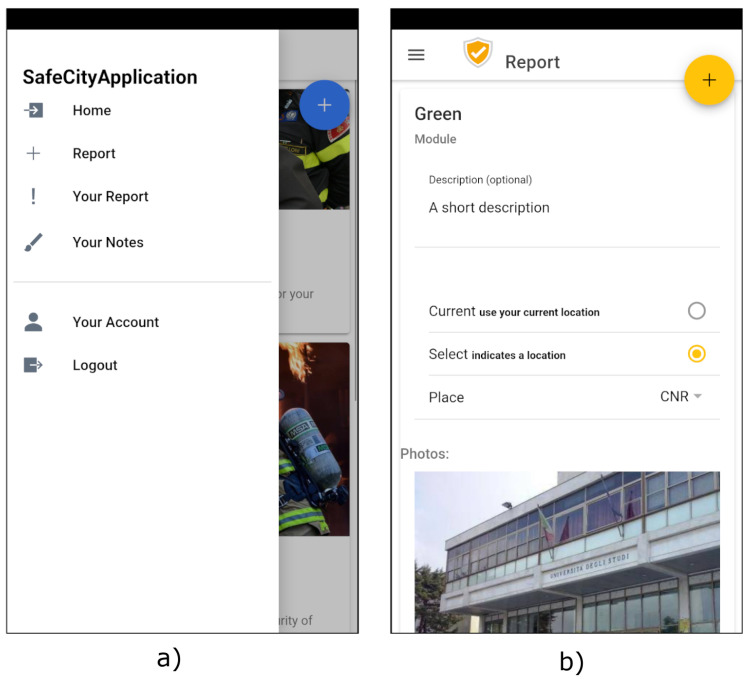
Screenshots of the Mobile Application. The menu of the application (**a**); the form used by the user to report a problem (**b**).

Another important component exposed by the developed system for the citizens is the Mobile Application that was developed through the Ionic Framework [47], an open-source mobile UI toolkit provided for building cross-platform apps [48]. It facilitates the creation of multi-platform Mobile Applications through the typescript programming language and HTML5 markup language. As the Mobile Application was implemented with the aim of supporting citizens to report problems in specific areas of the city, as shown in Figure 3a, the user can use the context menu to start a new report. Then, as shown in Figure 3b, she is guided in filling pre-defined surveys that also allow to attach photos and the GPS position of the zone in which the problem is detected. In addition, the app asks the user if she wants to share the report in anonymous or private mode. Thus, when the report is ready it is sent to the Cloud Server through the REST APIs exposed by the dedicated service running on the AWS platform to be then saved in the Firebase component. In addition, the Mobile Application is responsible for the subscription to the MQTT Broker exposed by the Cloud Server to receive the alerts. In our prototype the alerts are generated only by the Mobile Smart Monitoring Devices, but, due to the dynamic features of the MQTT publish/subscribe service, they are extendible to any other component of the system.

Moreover, the Front-end Application module was implemented through the Angular Framework [49], a very popular and widely used JavaScript platform for building responsive mobile and desktop web applications [50]. The Front-end Application is mainly responsible for the interactions with the Firebase database to acquire and show the real-time and historical data gathered through the Mobile Smart Monitoring Devices, and the history of the alerts generated in the whole system. In addition, it is connected to the MQTT Broker to receive real-time alerts and to send commands to the Mobile Smart Monitoring Devices to set or reset threshold for generating alerts. Thanks to the use of the Ionic and the Angular Frameworks and of the related plugins and libraries (e.g., Bootstrap [51]), the Front-end Application component respects the Usability transversal requirement because of its simple and intuitive interfaces.

Finally, the last element of the architecture is the Mobile Smart Monitoring Device. As shown in Figure 3 the Raspberry Pi 4 Model B board [34] was selected to implement this component in the first realized prototype; however, the modularity nature of the designed architecture allows to adopt other more suitable hardware solutions (e.g., an ad hoc designed hardware board). It is a small computer composed of multiple pins, called GPIOs, by which it is possible to control sensors and actuators.

**Figure 4:**
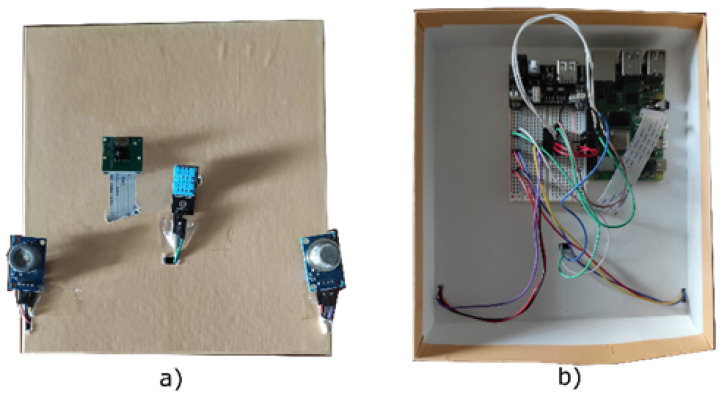
Developed prototype. The top of the realized prototype in which all the used sensors are exposed (**a**); the internal details of the realized prototype (**b**).

It is mainly responsible for the collection of monitoring information. In fact, as shown in Figure 4, several sensors have been connected to this device to detect temperature, gas values (e.g., CO, CO${}_{2}$, AMM), humidity, rain presence, and traffic conditions.

Particularly, the Raspberry was equipped with the following sensors and interfaces:

- MQ7 Sensor [27] and MQ135 Sensor [25] to monitor the air pollution through some parameters, such as carbon monoxide (CO), carbon dioxide, hydrogen, toulene, and ammonia. In general, the MQ sensors reveals the presence of gas in the air.
- The DHT11 Sensor to measure the temperature and the humidity.
- The YL-83 rain Sensor to detect rain.
- The Raspberry Pi Camera to acquire pictures of city streets and zones, and, consequently, in case of need, estimate the traffic conditions by querying the Cloud Server and executing the Python Flask machine learning algorithm.

Furthermore, the Mobile Smart Monitoring Device is also responsible for monitoring the received data and, in case of anomalies, generate an alert to be propagated through the MQTT Broker. Finally, it is also responsible for receiving commands to set or reset alert thresholds.

### Air Pollution Monitoring

To convert the analog output of MQ sensors (MQ7 and MQ135) into PPM values (the most used Air Quality Index) related to air pollution, it is necessary to carry out a calibration phase and define conversion function, as documented in the datasheets of each sensor [24,26]. The sensor output corresponds to the $R_{s}/R_{0}$ ratio which can be converted into PPM thanks to the following formula:

(1) $$PPM\left(log\right)=(\frac{\frac{R_{s}}{R_{0}}-y}{\mu })+x$$

where $R_{0}$ is the sensor resistance at 100 ppm CO in the clean air, while Rs is the sensor resistance at various concentrations of gases. Then, *y* is the numeric (not logarithmic) value along the ordinate axis of a known point A in the chart reported in the datasheet of the sensor, *x* the numerical value along the ordinate axis of the same chart, and μ the angular coefficient of the line. For more information about this formula and the specification of each sensor, it is possible to consult the corresponding datasheets of each sensor: MQ7 [26], MQ135 [24].

## 6. Functional Validation thorugh a Demo Session

To validate the developed prototype, two different types of validation were performed. The first one is the functional validation that is described in the present section while the performance evaluation is presented in the following section. For both the validations a demo session was organized at the University of Salento (Lecce, Italy). The campus of the University of Salento is big enough (1.20 Km × 0.70 Km) to let the authors use it as a “city district” with its “citizens”, represented by the students. Consequently, each designed functionality was simulated on the 20 July 2021. Three students were asked to install the Mobile Application and go around the campus to look for strange situations to be reported. Considering the nature of the functional tests aimed at demonstrating the feasibility of the system and its functionalities, the authors preferred to limit the number of involved testers to easily control and monitor the behavior of all the participants leaving the stress tests to the next performance validation described in the following section. As a result of the tests, two students reported the same strange situation (a bad smell near the Math department). Consequently, the “city official”, simulated by one of the authors, was able to test the generation of a notification related to the report and its visualization on the Front-end Application. By simulating the action of the city official, a Mobile Smart Monitoring Device was positioned in the selected area. Consequently, the area was monitored for 3 h, and no strange situation was revealed. However, to test all the other features, considering that the temperature was between 32 and 33 degrees Celsius and the system was set to send alerts only if the degree was higher than 35, the alert threshold for the temperature was set to 30. Therefore, some alerts were periodically sent, and it was verified that they were dispatched to all the system components, including the database. In addition, the traffic detector revealed that fifteen cars and twenty people passed in front on the sensors while only fourteen cars and eighteen people had actually passed. Finally, the functionalities related to the assignment of reports to the involvement of an operator was also tested by positioning a Mobile Smart Monitoring Device in the place in which the two citizens declared to perceive a strange smell.

## 7. Performance Validation

With the aim of testing the Cloud Server capacity in resisting to excessive loads, some stress tests were performed through the well-known JMeter application [52] that has been already used in various existing works presented in the literature (e.g., the work presented by Gajewski et al. [53]). As already discussed in the previous sections, different platforms and devices were exploited; therefore, in the first part of this section the details of each used platforms are discussed.

### 7.1. Details about Exploited Platforms

One of the platforms used within the present work is the IBM IoT Platform used to run the MQTT Broker by using the free plan called “Lite plan”. Then, the AWS services were exploited to run the described software on a virtual machine executed on an Amazon Elastic Compute Cloud (EC2) of which characteristics are reported in Table 5.

As already explained, on this virtual machine, the Cloud Server was run and it exposed some APIs to serve all the other components. To facilitate the interactions, both the “caching” and the “pageable” functionalities were introduced for, in turn, speed up reading operations and reduce the amount of sent information.

### 7.2. Performed Tests

According to Dhalla [54], we adopted a metric based on the average response time to evaluate the performance of our system. All tests were performed by using a notebook equipped with 16 Gb of RAM and an INTEL CORE i7 9th Gen processor. The computer was connected through a Wi-Fi access point included in a home optical fiber-based LAN which supports a speed connection up to 70 Gbit/s.

The tests were conducted by monitoring the most important interactions: the insertion of a report and the visualization of all the reports with the consequent assignment of one intervention to an operator. Such operations are the ones that require more resources due to the presence of images in the request. Their stress tests are important because the corresponding workload needed to address the request does not have to block any other functionality of the system (e.g., the notifications sent to the city officials or the receiving of new data from the sensors).

Consequently, the first category of stress tests was conducted by assuming that (a) 40,000 reports can be sent in an hour to the Cloud Server and that (b) such requests are linearly distributed over the analyzed hour. As already discussed in the previous paragraphs, when a citizen wants to submit a report, she has to use the Mobile Application that, through the exposed Graphical User Interface (GUI), guides her in the compilation of the pre-defined questions. Within the realized prototype, the survey was composed by two single questions regarding the category and the subcategory at which the report owns (e.g., report about the city, such as flooding, pothole, etc). To this aim, the Mobile application performed three main different actions in the background, corresponding to three different requests for the Cloud Server. The first one regarded the acquisition of the list of the report categories that the user can ask for, with the aim of showing the list in the GUI. The second request, instead, regarded the acquisition of the list of selectable subcategories and, lastly another request was dedicated to actually send the report. Table 6 reports the results obtained by monitoring this chain.

As shown by the table, it can be observed that the average time required to obtain the reports’ categories is higher than the time required to send a report. It is mainly related to the presence of images in the category list returned as a response by the system. Meanwhile, the request sent to submit a report usually consists only of the description and the identifier of the report’s category. In addition, the method invoked to insert a new report only invokes a single INSERT query against the database, conversely, the request to get the categories invokes a SELECT query which needs time to extract the requested data.

On the other hand, three other functionalities were tested: the reports visualization, the definition of an intervention, and the assignment of a report to an operator. Specifically, when a city official would like to intervene to solve a reported issue, she has to use the Front-end application to select an existing and unresolved report to, then, open a new “intervention” session and finally assign it to the operator. To this aim, some requests have to be sent to the Cloud Server through the dedicated REST API: the first one is used to obtain the list of all the existing reports; the second one is needed to open an “intervention”; and, finally, the third one regards the assignment of the intervention to an operator. This chain was monitored during a stress test that led to submit a total of 2000 simultaneous assignments in one hour. The obtained results are reported in Table 7.

Both experiments’ results demonstrate the complete feasibility of the system: when a huge number of requests is sent to the developed service, it continues to perform at least 11 operations per seconds in the first case and 1 operation in the second case. In addition, the network seems not to be a bottleneck for similar use cases and systems. As shown in the tables, the GET request for obtaining the reports is faster than the POST request needed to assign an agents. This is mainly caused by the higher complexity of the business logic invoked by the second method that is more complex than the one invoked by the first one. In fact, when the POST request arrives to the server, it has, at first to verify if an agent is available and such a task is the most time consuming one. The GET request, instead, is limited only by the extraction of the latest reports from the database that, in the worst case, consists only in the application of some filters based on the status. Finally, by comparing the “HTTP GET REPORTS” request with the “OPEN SESSION” one it is possible to notice that the second is faster than the first one as expected. In fact, the first request includes the transmission of data, while the second one only needs to identify the session opening before replying.

## 8. Discussion

As already discussed in the Related Works, the monitoring of environmental parameters through IoT technologies has been treated by different works present in the literature; however, as shown in Table 8 which highlights the main differences among our work and the existing ones, the already proposed solutions are mainly focused on the benefits of the technologies themselves or on the benefits of exploiting user feedback without any specific reference to the effectiveness of the combination of distributed mobile monitoring devices with citizens’ opinions.

The positive effects of this combination have, instead, been treated in our paper. The proposed solution, in fact, provides an ecosystem able to support citizens that want to share their feelings and city officials, who would like to have an up-to-date overview of the city conditions. The traditional IoT system already provided solutions to this aim, but the lack of correlation among user feedback and real-time data arriving from the sensors still gives rises to citizen complaints about their city’s conditions. Existing works usually propose solutions to improve the receipt of data or introduce automatic solutions to intervene in case of problems detection (e.g., turn on or off a HVAC system). However, to the best of our knowledge, none of them tries to combine feedback and real-time data to speed up the detection of strange situations and, furthermore, help city officials in recognizing events that sensors could ignore or that, more frequently, other cases can hide.

For this reason, in this paper, two realistic scenarios were introduced to help readers in understanding such an improvement with respect to traditional IoT systems. Such an inadequacy in solving the scenarios’ situations of existing works is highlighted in the comparison reported in Table 5, which summarizes the main differences among the present work and the works reported in the Related Works section.

If, for instance, the works proposed by Spandana et Shanmughasundram [3], or by Becnel et al. [2], Patil et al. [14], or also Garzon et al. [15], are taken into account, they can detect any anomaly present in different parts of the city with innovative increased granularity, but no city official would be able to investigate the situation detected by the user of the first scenario.

On the other hand, the works that are mainly focused on the monitoring of the city through the acquisition of user feedback without the adoption of IoT sensors, cannot address all the issues described in the reported scenarios. Indeed, neither the works proposed by White et al. [16], nor the ones proposed by Cecconi et al. [17] or Ertiö et al. [18] can, for instance, understand if the situation reported by the user of the first scenario is real or not.

Instead, a final consideration is necessary on the works proposed by Rinaldi et al. [19] and Tagliabue et al. [20], the one that, by applying an approach similar to the one presented in this paper to different situations (indoor monitoring), actually validate our approach. Although their automatic intervention in the mitigation of revealed situations cannot be directly applied to cities (i.e., no HVAC is appliable to a city) and, anyway, would limit the potentialities of direct interventions of city officials, in fact, their works already laid the basis for the exploitation of similar solutions in smart cities like the one proposed in the present paper.

As a demonstration of these potentialities, the infrastructure designed in this paper was implemented in a prototype that was used as a testbed for highlighting the effectiveness and the feasibility of the proposed approach. However, the exploitation of the solution in a real testbed was essential to also identify the drawbacks of the system and guided the authors in the definition of some lessons learned.

At first, indeed, through the analysis of the demo session results and the corresponding performance validation, we demonstrated the effectiveness of predefined surveys with respect to open questions. Even though, in fact, open questions are preferred by users, the predefined surveys facilitate and speed up the acquisition of the data and their interpretation.

In addition, the availability of some mobile smart monitoring devices can facilitate the fast intervention of authorities that, without a similar instrument already connected to the monitoring platform would need to (a) contact external companies to ask for a new detection through their systems, (b) wait for a reply and a monetary proposal, (c) decide which is the best proposal, (d) assign the task to the chosen company, (e) and wait for the availability of the needed equipment.

Finally, the combination of user feedback and parameters monitoring helps in the fast identification of critical situations and in facilitating the long-term decisions that are supported, in this way, by historical data on which anyone can leverage.

## 9. Conclusions

One of the main challenges of the past years and decades for local governments has been regarding citizens’ satisfaction with respect to city livability. One of the parameters usually exploited to monitor such a livability is the air quality that is increasingly gaining interest in societies in every part of the world also due to the negative effects that the global warming is causing.

For this reason, governments have started to look forward the adoption of new technologies and tools that can help them, first in monitoring their cities and then in adopting new strategies to mitigate the most impacting situations.

However, although great strides have already been made, citizens continue to reveal and report bad conditions in the areas in which they live.

With the aim of supporting city officials in both collecting such feedback from the citizens and exploit them in deciding if and where it is the case to further investigate or to invest money, this paper proposed an IoT solution to support (a) the collection of users reports, (b) the monitoring of specific zones of the city to effectively verify the reported conditions through the dynamic re-location of mobile devices, (c) differentiating interventions (e.g., traffic restrictions) per area, and (d) taking decisions about the position of new public green areas. Thanks to the developed system, city officials can exploit a centralized interface on which all the data and feedback are collected and, by its consultation, they can promptly and easily have an overview of the current situation of the city. In addition, the possibility of receiving notifications and dynamically re-locate mobile sensors to investigate reported anomaly situations allow them to intervene without the common delay foreseen by existing systems.

Therefore, with the further aim of demonstrating the potentialities of the proposed approach, a prototype was used as a testbed for highlighting the effectiveness and the feasibility of the proposed solution. Finally, the performance and functional validation allowed the definition of some lessons learned. As already discussed, the main contribution of the presented solution resides in the combination of distributed mobile monitoring devices with citizens opinions and feedback, that, as demonstrated, is greatly important to enhance both the citizens and city official roles. The presented approach and the developed solution can be used as an inspiration for other researchers that will work in the field to identify the best strategies, the best tools, and also the best approach to help both citizens and governments in the difficult task of mitigating pollution effects, through the combination of IoT technologies implemented in mobile distributed devices and the user feedback sent via a dedicated interface. Furthermore, the presented work opens to various future works that could better investigate the best way of collecting user feedback, for instance, through the Design Thinking approach [55] that could introduce the right tools and methodologies for guiding the design of each component of the system. In addition, future works would also investigate on the possibility of organizing workshops and live sessions in which the system will be exploited and evaluated by more users and in more participated demo sessions.

## Figures and Tables

**Table 1:** Functional requirements.

Requirement n.	Requirement Description
R1	Citizens would like to have the possibility of reporting strange situations about pollution revealed through their sensors.
R2	Citizens would like to receive notifications about strange pollution situations or intense traffic and understand if the administrations are intervening in any way.
R3	City officials would like to receive reports from citizens.
R4	City officials would like to receive notifications about reports from citizens.
R5	City officials would like to access historic data to, for instance, evaluate the possibility of planting new trees.
R6	City officials would like to set or reset the threshold for the generation of alerts.
R7	City officials would like to access fine grained data about a zone in case of necessity.
R8	City officials would like to monitor other situations like traffic in a specific zone, in case of necessity.

**Table 2:** Non-Functional requirements.

Requirement n.	Requirement Description
RN1	Security: The system should assure that all the manipulated data and the parts of the systems themself are protected against malware attacks or unauthorized access.
RN2	Usability: The system should expose user-friendly interfaces to improve user experience and facilitate the exploitation of exposed services.
RN3	Performance: The system should guarantee the reactivity of the system to user requests and interactions, in order to guarantee low waiting time before targeted operations’ execution and responses.
RN4	Reliability, Availability, and Maintainability: The system should be able to work and expose services without failures, guaranteeing, at the same time, minimum recovery time.
RN5	Scalability: Considering the mobile nature of the proposed system, this non-functional requirement is the most important one that should be satisfied by the system to support the possibility of adding increasing number of sensors all over the city.

**Table 3:** REST API for reports, interventions, operators, and survey.

Action	Method	Path	Input Parameters
Get the list of reports	GET	/api/report	/
Get the list of possible report categories	GET	api/category/getAllCategories	/
Get the list of subcategories	GET	api/category/<category_id>/getSubCategories	- *category_id*
Insert a new report category	POST	api/category/insert	- name - timestamp
Insert an intervention associated to a report	POST	api/intervention/	- title - report_id - description - timestamp

**Table 4:** REST API for the Machine Learning algorithm.

Action	Method	Path	Input Parameters
Get estimation	POST	/api/imageProcessing/estimation	- image

**Table 5:** Details of the Machine used on the Amazon Elastic Compute Cloud (EC2).

CPU	vCPUs	RAM (GiB)	Network Burst Bandwidth (Gbps)	EBS Burst Bandwidth (Mbps)
Intel Xeon Platinum 8000 (with a sustained all core Turbo CPU clock speed of up to 3.1 GHz)	2	4	5	Up to 2085

**Table 6:** Performance evaluation for submitting a report.

Request	#Samples in 1 h	Response Times (ms) Average	Throughput Transactions/s	Network (KB/s) Received	Network (KB/s) Sent
HTTP GET categories	40,000	232.38	11.11	10.59	4.8
HTTP GET subcategories	40,000	112.49	11.11	10.7	5.1
HTTP GET reports	40,000	110.54	11.11	15.81	7.2

**Table 7:** Performance evaluation for assigning an intervention to an operator.

Request	#Samples in 1 h	Response Times (ms) Average	Throughput Transactions/s	Network (KB/s) Received	Network (KB/s) Sent
HTTP GET REPORTS	2000	344.6	1.11	11.41	0.52
HTTP OPEN SESSION	2000	117.11	1.11	1.68	0.74
HTTP SEND AGENT	2000	448.31	1.11	0.43	0.66

**Table 8:** Comparison of related works and our solution.

	Multiple Mobile IoT Devices	Notifications to Citizen about Pollution	Exploit User Feedbacks	Indoor/Outdoor
Spandana et Shanmughasundram [3]	Mobile	No	No	Indoor
Becnel et al. [2]	Distributed	No	No	Indoor
Patil et al. [14]	Distributed	Yes	No	Indoor
Garzon et al. [15]	Distributed	Yes	No	Indoor
White et al. [16]	Fixed	No	Yes	Indoor
Re Cecconi et al. [17]	Fixed	No	Yes	Outdoor
Ertiö et al. [18]	Fixed	NO	Yes	Outdoor
Rinaldi et al. [19]	Distributed	Yes	Only data from sensors	Indoor
Tagliabue et al. [20]	Distributed	Yes	Yes	Indoor
Our solution	Mobile	Yes	Yes	Outdoor
